# MicroRNA-375 suppresses human colorectal cancer metastasis by targeting Frizzled 8

**DOI:** 10.18632/oncotarget.9811

**Published:** 2016-06-03

**Authors:** Lingling Xu, Tao Wen, Zhe Liu, Feng Xu, Lei Yang, Jian Liu, Guosheng Feng, Guangyu An

**Affiliations:** ^1^ Department of Oncology, Beijing Chao-Yang Hospital, Capital Medical University, Beijing 100020, China; ^2^ Medical Research Center, Beijing Chao-Yang Hospital, Capital Medical University, Beijing 100020, China

**Keywords:** colorectal cancer, microRNA-375, metastasis, FZD8

## Abstract

microRNAs are aberrantly expressed during the development and progression of a variety of human cancers, including colorectal cancer (CRC). Of these microRNAs, microRNA-375 (miR-375) was previously observed to be downregulated in human colorectal cancer(CRC) plasma and tissues, but its functions are largely unknown. Here, we investigated the impact of miR-375 on CRC metastasis. Specifically, miR-375 expression was significantly decreased in human CRC tissues compared with their matched noncancerous tissues (NCTs), and low levels of miR-375 predicted tumor metastatic potential. The up-regulation of miR-375 suppressed colorectal cancer cell migration and invasion in vitro and reduced tumor metastases in murine models established by both orthotopic implantation and spleen injection. Furthermore, we identified Frizzled 8 (FZD8) as a direct target of miR-375 in CRC, and miR-375 negatively regulated Wnt/β-catenin signaling by suppressing FZD8. More importantly, FZD8 expression inversely correlated with overall survival in human CRC patients and is a likely independent predictor of survival. Therefore, we concluded that miR-375 functions as a tumor-suppressive microRNA by directly acting upon FZD8, which may serve as a new therapeutic target to inhibit tumor metastasis in CRC.

## INTRODUCTION

Colorectal cancer (CRC) is the third most common cancer and one of the leading causes of cancer-related death worldwide [1]. The five-year survival rate of patients with resectable CRC has recently improved, exceeding 90%, but the survival rate of CRC patients with unresectable metastases remains discouraging at less than 10% [2]. Notably, approximately half of CRC patients develop distant metastases, especially liver metastases, which are the main cause of death in patients [3]. Tumor metastasis is a complex process that consists of multiple sequential steps, including the invasion of cancer cells into surrounding tissues, intravasation, survival in circulation, arrest at distant organ sites, extravasation, and growth in distant organs [4]. Despite considerable advances over the past decades, the molecular mechanisms underlying tumor metastasis have been less understood.

MicroRNAs (miRNAs), a class of small non-protein-coding RNAs, have been identified as novel gene expression regulators that bind the 3′ untranslated regions (UTR) of target mRNA, thereby resulting in mRNA degradation or the blockade of mRNA translation [5]. miRNAs are essential for the regulation of many biological processes, such as the cell cycle, proliferation, differentiation and apoptosis [6–8]. Moreover, accumulating evidence has implicated miRNAs in various pathological conditions, including cancer [9, 10]. Deregulated miRNAs have been found to be associated with tumor initiation, promotion and progression by acting on many oncogenes and tumor suppressors. Furthermore, they have been correlated with disease stage, metastasis and survival in numerous human cancers, including CRC [11]. Specifically, some miRNAs that act as tumor suppressors are downregulated in tumorigenesis, whereas other miRNAs are over-expressed in cancer tissue compared with normal tissues and act as tumor promoters [12, 13]. Among these miRNAs, miR-375 has recently been documented to be downregulated in various types of cancers. For example, several studies reported that pancreatic miRNA-375, which directly targets PDK1, plays key roles in the glucose regulation of insulin gene expression and β-cell growth and was evidently downregulated in pancreatic carcinoma [14, 15]. We and other groups have found that the down-regulation of miR-375 is pronounced in the plasma and cancer tissues of colorectal cancer patients [16, 17]. However, the contribution of this down-regulation to the development and progression of CRC remains unknown, and the related mechanisms and functions of miR-375 in CRC are yet to be determined.

In this study, we aimed to explore the clinical implication of miR-375 in CRC and the underlying mechanisms through in vivo and in vitro investigations. We found that miR-375 was not only markedly downregulated in human CRC tissues but could also predict the metastatic potential of CRC patients. Moreover, the up-regulation of miR-375 suppressed colorectal cancer cell migration and invasion in vitro and reduced tumor metastases in murine models established with both orthotopic implantation and spleen injection. We further verified that Frizzled 8 (FZD8) is a direct and functional target of miR-375, and its overexpression is associated with decreased survival in CRC patients. Because FZD8 is a key receptor for the initiation of Wnt/β-catenin signaling pathway [18], which is well known for its role in the development and promotion of cancer metastasis, we investigated the ability of miR-375 to regulate Wnt/β-catenin signaling by inhibiting FZD8 expression. Our findings provide novel insights into the functions and clinical relevance of miR-375 in CRC and suggest that miR-375 and FZD8 may be used as novel prognostic markers and potential therapeutic targets in clinical practice.

## RESULTS

### Low expression of miR-375 predicts the metastatic potential of human colorectal cancer

**Figure 1:**
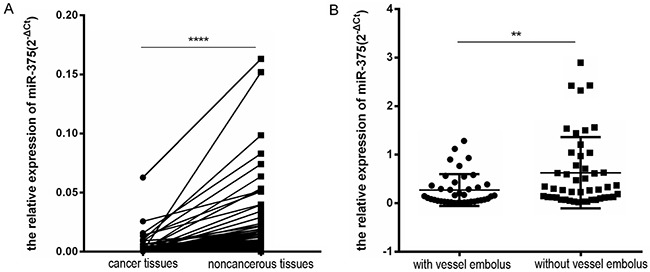
Low miR-375 expression predicts metastatic potential in human colorectal cancer (CRC) patients **A.** miR-375 expression was measured in 90 paired human CRC and adjacent noncancerous tissues (NCTs) by quantitative reverse transcription polymerase chain reaction (qRT-PCR). Two–tailed non-parametric Wilcoxon test were used to evaluate the statistical differences of miR-375 levels. Specifically, miR-375 expression was markedly downregulated in cancer tissues compared with their corresponding NCTs. U6 small nuclear RNA was used an as internal control (****p <0.0001). **B.** CRC patients with vessel emboli (n=41) expressed lower levels of miR-375 than patients without vessel emboli (n=49) (**p=0.004), indicating that miR-375 expression may inversely correlate with the metastatic potential of CRC patients.

We examined the miR-375 levels in all 90 pairs of human colorectal cancer tissues and their corresponding noncancerous tissues (NCTs) by qRT-PCR. As shown in Figure 1A, miR-375 expression was distinctively downregulated in colorectal cancer tissues relative to their matched NCTs (*p*<0.0001). Among the samples from 90 colorectal cancer patients, 61 cases(67.7%) exhibited a >50% reduction in miR-375 expression compared with their NCTs. On average, the miR-375 levels were decreased 2- to 3-fold in human colorectal cancer tissues relative to their NCTs. Furthermore, we observed that miR-375 expression in CRC was not associated with gender, age, differentiation, stage, metastases or perineural invasion (all *p*>0.05) but correlated well with vessel embolus (*p*=0.004, Supplementary Table S1). In contrast, the median level of miR-375 in patients without vessel embolus was 0.3, whereas the median level in patients with vessel embolus was 0.1, suggesting that patients expressing low levels of miR-375 were more likely to develop a vessel embolus (Figure 1B, *p*=0.004). Because vessel emboli have been associated with an increased incidence of tumor metastasis (especially liver metastasis for CRC) and an overall decrease in the survival rate [19–20], we investigated the relationship between low miR-375 expression and CRC metastasis.

### miR-375 suppresses CRC cell migration and invasion in vitro

**Figure 2:**
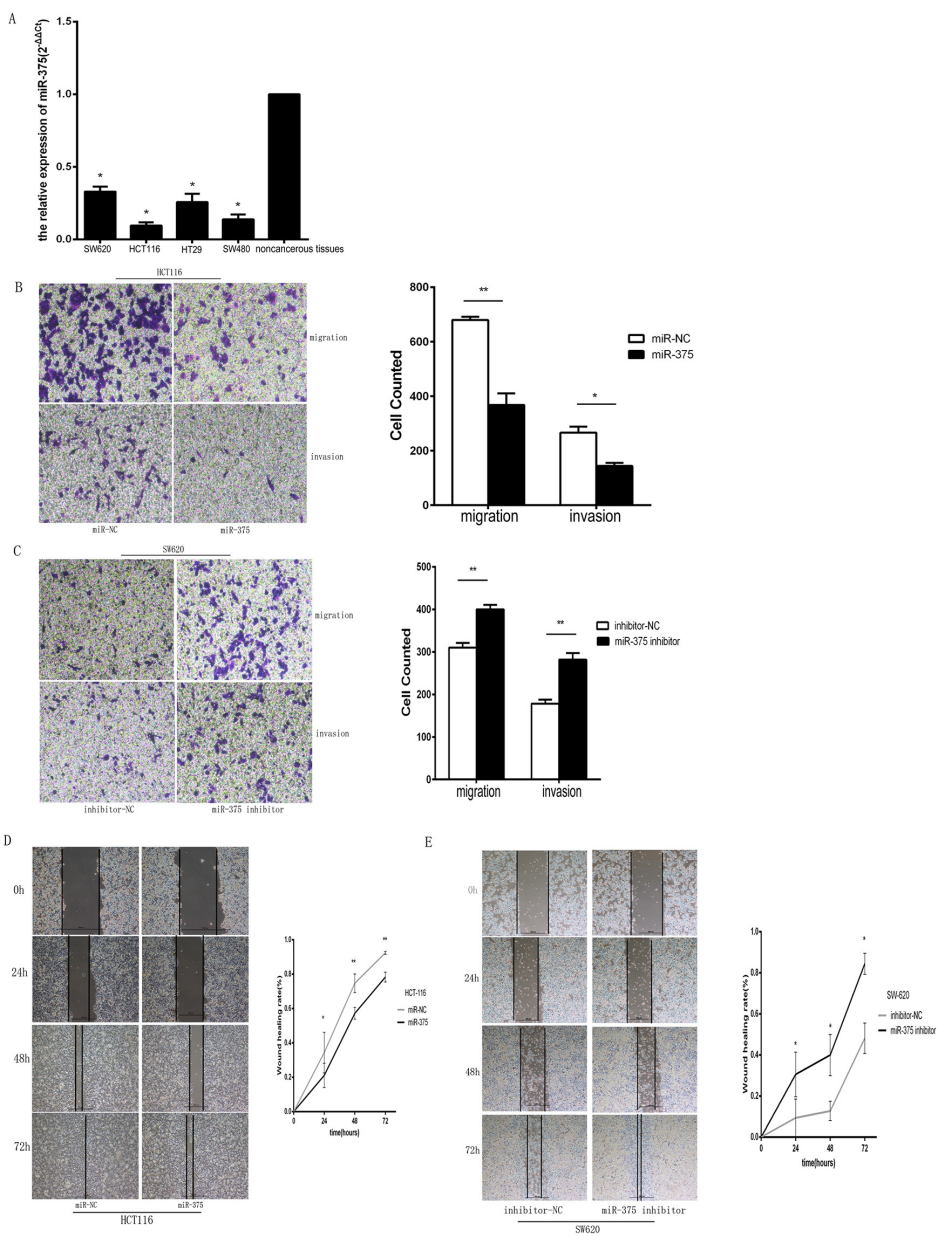
miR-375 suppresses CRC cell migration and invasion in vitro **A.** Real-time PCR analysis of the relative expression levels of miR-375 in four CRC cell lines (HCT116, HCT29, SW480, and SW620) and noncancerous tissues (NCTs). The error bars represent the mean±standard deviation (SD) of 3 independent experiments. Specifically, miR-375 expression was lowest in HCT116 cells, whereas SW620 cells expressed relatively high levels of miR-375 (* p<0.05). **B.** The migration and invasiveness of HCT116 cells were analyzed after transfection with miR-375. The indicated migrating or invading cells were quantified in 5 random fields after culture in Matrigel-coated or non-coated Transwell assays, respectively. The overexpression of miR-375 markedly suppressed the invasiveness and migration of HCT116 cells compared with the control group (vs. miR-NC vector; p=0.009 and 0.02, respectively). **C.** The migration and invasiveness of SW620 cells after transfection with miR-375 inhibitor were also investigated. The down-regulation of miR-375 significantly increased cell migration and invasion (vs. inhibitor-NC group; p=0.005 and 0.005, respectively). **D.** The wound-healing rate was calculated at0, 24, 48 and 72 hours after wounding. In HCT116 cells, the overexpression of miR-375 inhibited the migration rate of cells at 24, 48 and 72 hours compared with the control cells, which were transfected with miR-NC vector (p=0.040,0.005 and 0.008, respectively). **E.** In SW620 cells, knocking down miR-375 increased the migration rate at various time points (vs. the control cells receiving inhibitor-NC, p=0.020, 0.010 and 0.010, respectively). The experiments in A-E were repeated at least 3 times, and error bars represent the mean±SD. Original magnification, B and C, 200×.

We measured the levels of miR-375 in four CRC cell lines using qRT-PCR and found an approximately 11.8-fold decrease in the miR-375 levels in HCT116 cells, a 3.7-fold decrease in HT29 cells, a 7.1-fold decrease in SW480 cells, and a 3.2-fold decrease in SW620 cells relative to NCTs (Figure 2A). Therefore, we selected HCT116, which expressed the lowest levels of miR-375, to stably over-express miR-375 by plasmid transfection. The successful up-regulation of mature miR-375 was confirmed by qRT-PCR (Supplementary Figure S1). A transwell assay showed that miR-375 up-regulation drastically suppressed the invasiveness and migration of HCT116 CRC cells (Figure 2B). Moreover, we transiently transfected a miR-375 inhibitor into SW620 cells, which expressed relatively high levels of endogenous miR-375 (Supplementary Figure S2). As demonstrated in Figure 2C, the inhibition of miR-375 significantly increased cancer cell migration and invasion. Moreover, wound-healing assays showed that miR-375 up-regulation inhibited the rate of HCT116 cell migration, whereas miR-375 knockdown increased this rate in SW620 cancer cells (Figure 2D–2E). Collectively, these results suggest that miR-375 is a negative regulator of CRC metastasis.

### Up-regulation of miR-375 inhibits CRC metastasis in vivo

**Figure 3:**
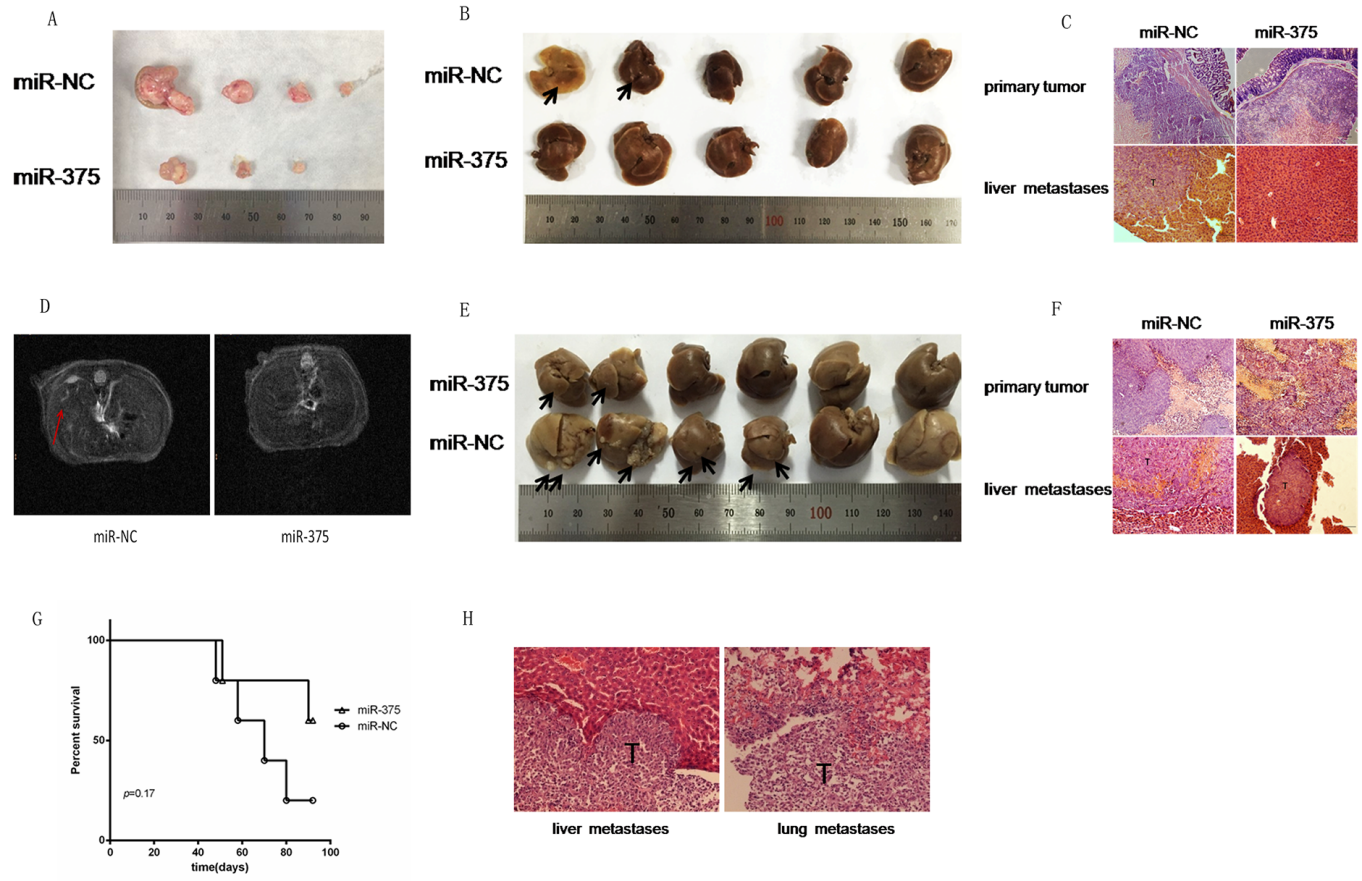
Up-regulation of miR-375 inhibits CRC metastasis in vivo **A.** Photographs of xenograft formation in mice implanted withHCT116 cells containing miR-375 or miR-NC vector. Twelve weeks after orthotopic implantation, the mice in both groups were euthanized to remove the xenografts. Xenograft formation was markedly reduced in mice bearing miR-375 HCT116 cells compared with mice bearing miR-NC vector cells. **B.** Twelve weeks after orthotopic implantation, the mice were euthanized to examine the livers for tumor metastases. Two liver metastases were found in the miR-NC group, but no metastases were found in the in miR-375 group. The arrows indicate surface metastatic nodules. **C.** The characteristics of tumor cells in the orthotopic xenografts and liver metastases were visualized by H&E staining. **D.** Representative MRI images of mice that had developed liver metastases after spleen injection. **E.** Six weeks after spleen injection, the mice in both groups were euthanized to collect their livers. The arrows indicate surface metastatic nodules. **F.** The characteristics of tumor cells in the spleen and liver metastases were visualized by H&E staining. **G.** Six-week-old male NOD/SCID mice were subcutaneously injected with HCT116 cells stably expressing the miR-375 or miR-NC vector, and the survival rates of the two groups of mice were calculated. Survival tended to be longer in the miR-375 group than in the control group. **H.** Liver metastases and lung metastases in the NOD/SCID mice, which were implanted with the indicated cells, were confirmed by H&E staining.

To further validate that miR-375 suppresses CRC metastasis, as described above, we established an orthotopic implantation murine model to represent tumor growth and metastasis. As shown in Figure 3A, xenograft formation was drastically reduced in mice bearing miR-375 HCT116 cells compared with mice bearing miR-NC vector cells. More importantly, orthotopically implanted miR-375 HCT116 cells gave rise to fewer liver metastases than orthotopically implanted miR-NC cells (2 liver metastases for the miR-NC group vs. no metastases for the miR-375 group) (Figure 3B). H&E staining confirmed the characteristics of tumor cells in the liver metastases of the orthotopic implantation model (Figure 3C).

In addition, we established a liver tumor metastasis murine model via spleen injection to imitate the colonization and outgrowth phase of the tumor metastatic cascade. We used MRI to dynamically detect liver metastases, which can be visualized 4 weeks after injection (Figure 3D). Compared with the miR-NC group, the number of total metastases, tumor volume and disease severity were lower in the miR-375 group (2 liver metastases for the miR-375 group vs. 4 liver metastases for the miR-NC group) (Figure 3E). The metastasis rate in the miR-375 group was 33.3%, whereas it was 66.7% in the miR-NC group. The miR-NC mice displayed prominent liver metastases, peritoneal metastases and ascites, whereas significant liver metastases, peritoneal metastases or ascites were not observed in the miR-375 group (Supplementary Figure S3-4, Supplementary Table S2). Likewise, H&E staining confirmed the characteristics of tumor cells in the spleen xenografts and liver metastases (Figure 3F). These results indicate that miR-375 is a key inhibitor that suppresses CRC tumor cell growth and metastasis in vivo.

Finally, HCT116 cells stably expressing miR-375 or miR-NC were subcutaneously injected into 2 groups of 6-week-old NOD/SCID mice to investigate the association between miR-375 and the survival of nude mice. After 3 months, we observed that the miR-375 group exhibited a tendency for longer survival than the miR-NC group (Figure G). The miR-375 group did not exhibit significant metastases, whereas the miR-NC group showed higher metastatic potential at various sites, including the lung, liver and peritoneum (no metastases for the miR-375 group vs. 1 liver metastasis,2 lung metastases and 2 peritoneal metastases for the miR-NC group) (Figure 3H, Supplementary Figure S5, Supplementary Table S3). Taken together, our data show that miR-375 functions as an important tumor suppressor in CRC by suppressing tumorigenesis and metastatic colonization.

### FZD8 is a direct target of miR-375 and is associated with poor prognosis in CRC patients

**Figure 4:**
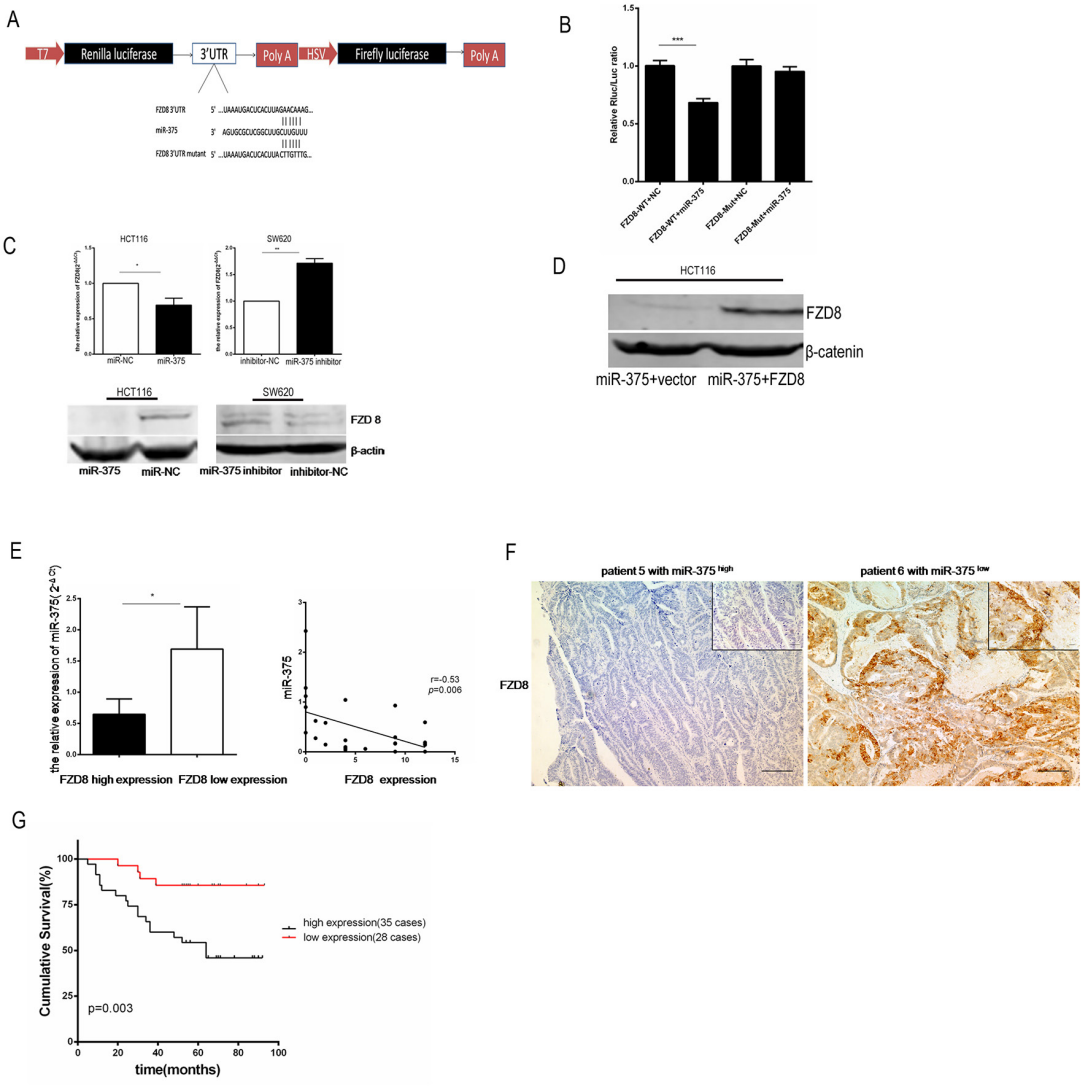
FZD8 is a direct target of miR-375 and is associated with poor prognosis in CRC patients **A.** Schematic of the human FZD8 3′-UTR luciferase constructs containing wild-type and mutant (FZD8 3′-UTR) miR-375 target sequences. **B.** HCT116 cells were co-transfected with the luciferase constructs and miR-375. The luciferase activities were measured 48 h after transfection using the Dual-Luciferase Reporter Assay System. **C.** PCR analyses showed that the ectopic restoration of miR-375 downregulated the mRNA levels of FZD8. The values are expressed as the mean±SD of 3 independent experiments. Western blot analyses showed that the ectopic restoration of miR-375 downregulated the expression of FZD8 protein. **D.** Changes in FZD8 expression in HCT116 cell bearing miR-375 in response to FZD8 transfection were analyzed by western blotting. All experiments were repeated at least three times. **E.** An inverse correlation was observed between FZD8 and miR-375 expression in CRC patients. The correlation between miR-375 and FZD8 was determined with linear regression lines, and the significance was assessed with a Spearman correlation (*p*=0.006). **F.** IHC staining of FZD8 in human CRC tissues. Representative images show higher FZD8 expression in human samples expressing low miR-375 levels and lower FZD8 expression in human samples expressing high miR-375 levels. **G.** The up-regulation of FZD8 was associated with poorer overall survival. Kaplan-Meier overall survival curve comparing 2 groups of CRC samples expressing high (greater than the median) and low (less than the median) FZD8 levels. The *p* value is based on a log-rank test.

Using target gene prediction and signal pathway analyses, we previously identified FZD8 as a likely target of miR-375 in CRC [17]. To verify this finding, we cloned the 3′UTR of the FZD8 gene into the luciferase construct pmiR-RB-REPORT (Figure 4A). The luciferase assay revealed that miR-375 reduced the activity of the luciferase reporter gene fused to the FZD8 3′-UTR in HCT116 cells by 63%±3.5% (*p*=0.0006) compared with miR-NC cells. In contrast, miR-375 did not decrease the luciferase activity of a mutant construct (Figure 4B). Likewise, PCR and western blot analyses confirmed that the ectopic restoration of miR-375 in HCT116 cells inhibited the expression of FZD8, whereas the knockdown of miR-375 in SW620 cells significantly elevated FZD8 expression (Figure 4C). We then performed a rescue experiment by co-transfecting HCT116 cells with miR-375and FZD8. The overexpression of miR-375 suppressed FZD8 expression in HCT116 cells, whereas the up-regulation of FZD8 inhibited this effect, thus confirming a direct association between miR-375 and FZD8 (Figure 4D).

The identified link between miR-375 and FZD8 in CRC cell lines led us to attempt to recapitulate this relationship in human CRC. To this end, we searched for a correlation between the miR-375 levels and the expression of FZD8 in human CRC tissues. We observed that the expression of miR-375 was inversely associated with FZD8 expression in 33 CRC patients (*p*=0.006, r=−0.53, Figure 4E). As shown in Figure 4F, the expression of FZD8 was higher in human tissue samples that expressed low miR-375 levels, whereas FZD8 was low in tissues that expressed high miR-375 levels. The tissue samples from BALB/C nude mice harboring subcutaneous xenografts also showed the same trend (Supplementary Figure S6). Similarly, the expression of FZD8 protein was upregulated in both liver and lymph node metastases compared with NCTs (*p*=0.001 and *p*=0.006, respectively, Supplementary Figure S7, Supplementary Table S4). Furthermore, the expression of FZD8 in human CRC tissues inversely correlated with overall survival (OS). High FZD8 expression correlated with shorter overall survival in CRC patients (*p* =0.003, Figure 4G). After adjusting for age, gender, differentiation, TNM stage, invasive depth, metastases and perineural invasion, multivariate analyses confirmed that FZD8 expression, lymph node involvement and vessel embolus were independent prognostic factors for CRC survival (Table 1). However, FZD8 expression was not significantly associated with the clinicopathological features of colorectal carcinoma (Supplementary Table S5). Taken together, these results suggest that miR-375 is inversely associated with FZD8, whose expression might serve as predictor of poor survival among human CRC patients.

**Table 1:** Univariate and multivariate analyses of FZD 8 expression and overall survival of CRC patients

Variables	Classification	Univariate analysis		Multivariate analysis	
		HR(95%CI)	*P*	HR(95%CI)	*P*
Age	>60/≤60	0.98(0.40-2.40)	0.96	-	-
Gender	male/female	1.89(0.74-4.84)	0.18	-	-
Differentiation	Poor+mucinous/moderate+high	1.60(0.54-4.74)	0.39	-	-
TNM stage	III+IV/I+II	3.45(1.49-8.02)	0.004^*^	-	-
Invasive depth	T3+T4/T1+T2	5.25(0.71-39.07)	0.11	-	-
Lymph nodes	Positive/negative	3.79(1.63-8.80)	0.002^*^	3.05(1.27-7.34)	0.01
Metastases	Positive/negative	4.61(1.51-14.11)	0.01^*^	-	-
FZD8 expression	High/low	4.46(1.51-13.19)	0.01^*^	3.55(1.17-10.79)	0.03
Perineural invasion	Positive/negative	2.53(0.98-6.48)	0.05	-	-
Vessel embolus	Positive/negative	4.85(1.85-12.71)	0.001^*^	3.19(1.13-8.99)	0.03

HR: hazard ratio; 95% CI: 95% confidence interval.

### miR-375 modulates the Wnt/β-catenin pathway by targeting FZD8

**Figure 5:**
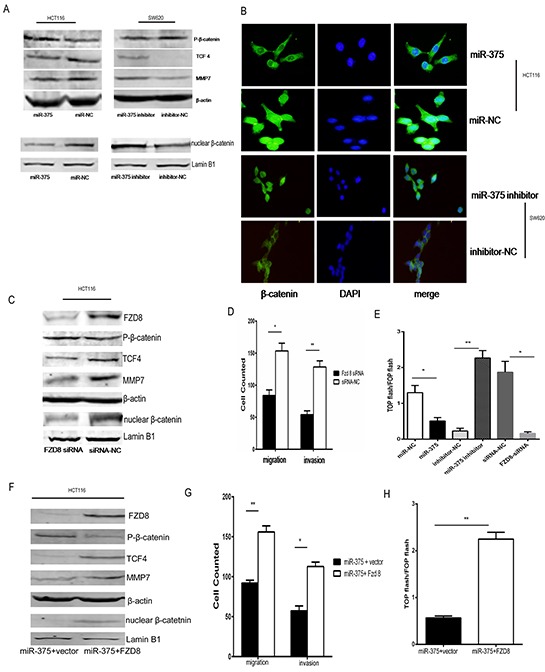
miR-375 modulates the Wnt/β-catenin pathway by targeting FZD8 **A.** Western blot analysis of phosphorylated β-catenin, TCF4, MMP7 and nuclear β-catenin protein expression response to deregulated miR-375 expression in the indicated cells. Nuclear fractions of the indicated cells were analyzed by western blotting. Lamin B1 was used as a loading control. **B.** Subcellular β-catenin localization in indicated cells was assessed by immunofluorescence staining. **C.** The knockdown of FZD8 in HCT116 cells with specific siRNA was confirmed by western blotting. The expression levels of phosphorylated β-catenin, TCF4, MMP7 and nuclear β-catenin in response to downregulated FZD8 were analyzed by western blotting. **D.** The migration and invasiveness of HCT116 cells were analyzed after transfection with FZD8 siRNA. **E.** Indicated cells transfected with TOP flash or FOP flash and Renilla pRL-TK plasmids were subjected to dual-luciferase assays 48 hours after transfection. Reporter activity detected was normalized by Renilla luciferase activity. **F.** Altered FZD8 expression in HCT116 cell bearing up-regulated miR-375 in response to FZD8 transfection and the expression of phosphorylated β-catenin, TCF4, MMP7 and nuclear β-catenin protein were analyzed by western blotting. All experiments were repeated at least three times. **G.** The migration and invasiveness of HCT116 cell bearing up-regulated miR-375 were analyzed after FZD8 transfection. The indicated migrating or invading cells were quantified in 5 random fields after culture in matrigel-coated or non-coated transwell assays, respectively. **H.** Luciferase-reported TCF/LEF transcriptional activity in indicated cells. The experiment was repeated at least three times, and error bars represent the mean±SD. * p<0.05,**p<0.01.

Considering the canonical role of the Wnt/β-catenin pathway in tumorigenesis and metastases and because FZD8 is an upstream receptor in the canonical Wnt/β-catenin signaling pathway [21], we hypothesized that miR-375 similarly inhibits the Wnt/β-catenin pathway. As shown in Figure 5A, the levels of TCF4, MMP7 and nuclear β-catenin were downregulated by the ectopic restoration of miR-375 in HCT116 CRC cells. Accordingly, the level of phosphorylated β-catenin was upregulated (Figure 5A). Likewise, immunofluorescence staining showed that the overexpression of miR-375 reduced the nuclear accumulation of β-catenin in HCT116 CRC cells, which is an important feature of the activation of Wnt/β-catenin signaling (Figure 5B). Conversely, the transfection of miR-375 inhibitor in SW620 CRC cells upregulated the expression of TCF4, MMP7 and nuclear β-catenin and downregulated the expression of phosphorylated β-catenin protein. The nuclear translocation of β-catenin was also activated in the miR-375 inhibitor group.

To further verify that FZD8 is a key factor in the miR-375-mediated regulation of Wnt/β-catenin pathway, we used specific siRNAs against FZD8 to knockdown FZD8 expression in HCT116 cells. We found that FZD8-siRNA significantly reduced the expression of FZD8 protein and subsequently inhibited the levels of TCF4, MMP7 and nuclear β-catenin, whereas it upregulated the expression of phosphorylated β-catenin protein (Figure 5C); these effects recapitulated those of the overexpression of miR-375. Functional assays showed that the down-regulation of FZD8 inhibited HCT116 cell migration and invasion (Figure 5D), which resembled the inhibitory effects of miR-375 overexpression on cells described above. As expected, miR-375 overexpression and FZD8-siRNA decreased the transactivating activity of β-catenin in HCT116 cells, whereas miR-375 inhibitor increased the transactivating activity of β-catenin in SW620 cells, as determined by a β-catenin reporter assay (Figure 5E).

Additionally, we performed a rescue experiment by co-transfecting HCT116 cells with miR-375 and FZD8. As expected, a western blot analysis demonstrated that FZD8 reversed the miR-375-mediated inhibition of TCF4, nuclear β-catenin, and MMP7 and upregulated phosphorylated β-catenin protein (Figure 5F and Supplementary Figure S8). Strikingly, the reductions in CRC cell migration, invasion and TCF/LEF transcriptional activity caused by miR-375 overexpression were effectively reversed by FZD8 (Figure 5G–5H). Collectively, these findings suggest that FZD8 is an essential functional mediator of miR-375-repressed cell migration and invasion and that miR-375 regulates the Wnt/β-catenin pathway by targeting FZD8 in CRC.

## DISCUSSION

Cancer invasion and distant metastasis, which are complex, multistep processes that are likely controlled by various genetic and/or epigenetic changes, are the leading causes of more than 90% of cancer-related deaths, including CRC deaths [22]. However, the regulatory factors that are responsible for molecular changes that initiate metastatic progression have not been defined. The identification of the upstream regulators of metastasis appears to be essential for a better understanding of cancer metastasis and subsequent therapeutic targeting. Recent studies have highlighted the roles of miRNAs in a broad range of developmental processes associated with tumorigenesis and metastasis [23, 24]. miRNAs have been found to be able to suppress multiple target genes and thereby modulate multiple steps of metastatic progression in various cancer types [25, 26] In the context of CRC, some miRNAs have been discovered to regulate different cellular behaviors that control invasion and metastasis, and their roles remain controversial. For example, miR-625, miR-103/miR-107, miR-21 and miR-301 have been found to promote CRC to invade and metastasize by stimulating multiple metastasis-promoting genes [27–30], whereas miR-99, miR-137, miR-132 and miR-128 function as tumor suppressors to inhibit the metastasis of CRC [31–34]. In the current study, we, for the first time, identified miR-375 as a novel metastasis inhibitor of CRC with clinical relevance. Indeed, miR-375 was previously found to be restricted only to pancreatic islets, but it has since been revealed to have important functions in tumorigenesis [14, 35]. Several in vitro and in vivo studies have shown that pancreatic miRNA-375 directly targets PDK1, plays key roles in the glucose regulation of insulin gene expression and β-cell growth and is down-regulated in pancreatic carcinoma [15]. Recently, Dai et al reported that miR-375 expression is frequently down-regulated in colorectal cancer tissues compared with the non-tumor counterparts [36]. We also demonstrated that the level of miR-375 was significantly decreased in the plasma of CRC patients and correlated well with the expression observed in tissue samples, suggesting that miR-375 may serve an alternative biomarker of minimally invasive CRC [17]. However, the molecular mechanisms related to the downregulation of miR-375 in CRC have not been fully studied. miR-375 has been suggested to inhibit colorectal cancer growth by targeting the PI3K/Akt signaling pathway [37] and reduce cell viability by targeting YAP1 to induce apoptosis [38]. However, possible other functions of miR-375 have not yet been identified. Notably, our extensive analysis of clinical samples showed that low miR-375 expression was not only prominent in the tissues of CRC patients but also associated with metastatic. Thus, we investigated miR-375 as a clinically relevant regulator of CRC metastasis. We first confirmed that up-regulating miR-375 in vitro suppressed CRC cell migration and invasion, whereas miR-375 knockdown in colorectal cancer cells promoted their migration and invasion. Additionally, we adopted two murine metastasis models to deeply explore the role of miR-375 in the metastasis of CRC. The complex invasive-metastatic cascade can be conceptually divided into two major phases: the translocation of cancer cells from the primary tumor to a distant organ and the colonization of a secondary organ and outgrowth of these translocated cells [39]. We established an orthotopic implantation model to mimic the first phase of the tumor metastatic cascade and used a spleen injection model to theoretically imitate the colonization and outgrowth phase of the tumor metastatic cascade. Our results showed that the overexpression of miR-375 diminished the number of liver metastases in both models. Collectively, these in vitro and in vivo results suggest that miR-375 plays an anti-metastatic role in CRC, which provides new insights into the functions of miR-375 in the development and progression of CRC.

We next investigated the molecular mechanisms by which miR-375 regulates metastasis in CRC. For this purpose, we predicted likely targets of miR-375 using bioinformatics and identified FZD8, a member of the Frizzled (FZD) family, as a direct target of miR-375. The overexpression of miR-375 suppressed FZD8 expression in CRC cell lines, whereas the up-regulation of FZD8 antagonized the suppressive effect of miR-375, which confirmed a direct interaction between miR-375 and FZD8. Additionally, miR-375 was negatively associated with the FZD8 expression levels in human CRC tissues. Whereas miR-375 expression was low, FZD8 expression was drastically elevated in CRC patients, in liver and lymph nodes metastases. Furthermore, we found that patients expressing high FZD8 levels had shorter overall survival than patients expressing low FZD8 levels. Thus, FZD8 might serve as an independent prognostic marker for CRC patients.

The cellular signaling mechanisms underlying the metastasis of CRC have been extensively studied. Several classical pathways, including the Wnt/β-catenin, PI3K/Akt, and TGF-β pathways, have been found to be aberrantly activated and play vital roles in the development and progression of CRC [40–42]. Among these pathways, Wnt/β-catenin signaling is known to play a major role in the promotion of CRC metastasis [43] and correlates with poor clinical prognosis [44]. Hu et al found that several factors, such as stromal cell-derived factors, can induce the Wnt/β-catenin pathway to promote CRC progression [45], whereas Wang et al showed that Brg-1 can inhibit the Wnt/β-catenin pathway to suppress CRC metastasis [46]. Moreover, nuclear β-catenin accumulation, another important feature of Wnt/β-catenin signaling activation, has frequently been observed in primary CRC tissues [40], which further highlights the essential role of Wnt/β-catenin in the process of metastasis. Notably, FZD is known as an upstream receptor implicated in the canonical Wnt/β-catenin signaling pathway [18, 21]. Wnt/β-catenin signaling is initiated when Wnt ligands bind to FZD members, including FZD8[47]. For example, FZD8 has been shown to regulate the canonical Wnt/β-catenin pathway in arthritis synovial fibroblasts and alveolar epithelial cell trans-differentiation in rat models [48, 49]. Moreover, FZD8-mediated Wnt/β-catenin signaling appeared to participate in mediating resistance to chemotherapy in triple-negative breast cancer [50]. Therefore, we speculated that miR-375 may inhibit Wnt/β-catenin signaling pathway by suppressing its direct target-FZD8 to regulate the metastasis of CRC. As anticipated, our data showed that the overexpression of miR-375 significantly inhibited the Wnt/β-catenin pathway and downregulated FZD8, which consequently decreased cancer cell invasion and metastasis. Accordingly, the restoration of FZD8 expression neutralized the inhibition of Wnt/β-catenin signaling by miR-375.

In summary, the results presented herein show that miR-375 exerts anti-metastatic effects during the progression of CRC. miR-375 may function as an important negative regulator of the Wnt/β-catenin pathway by targeting FZD8. We suggest that miR-375 may be clinically useful for developing a new prognostic biomarker and therapeutic target for CRC metastasis.

## MATERIALS AND METHODS

This study was approved by the Clinical Research Ethics Committee of Beijing Chao-Yang Hospital, Capital Medical University, Beijing, China. Informed consent was obtained for each patient. The clinical data were prospectively collected for all involved participants.

### Human samples and cell lines

A total of 90 pairs of human primary colorectal cancer tissues and their adjacent normal tissues were collected from December 2013 to October 2015. Patients recruited in this study were newly diagnosed with CRC and had not received any treatment. Pathological analyses were used to confirm the diagnosis, and the patients were staged according to the tumor-node-metastasis (TNM) staging system of the International Union Against Cancer. The tumor and adjacent normal tissues were obtained after surgical resection and immediately placed in liquid nitrogen for further analyses. In addition, a total of 63 paraffin-embedded human CRC specimens that were histopathologically diagnosed were collected from January 2007 to October 2015. The clinical information of patients with CRC is shown in Supplementary Tables S1 and S4. Four human colorectal cancer cell lines, HCT116, HCT29, SW480, and SW620, were obtained from the American Type Culture Collection (ATCC). HCT116 and HCT29 cells were cultured in McCoy's 5A medium, and SW480 and SW620 were maintained in Leibovitz's L-15 medium. All media (Invitrogen) were supplemented with 10% FBS(Gibco). The cells were cultured under the conditions recommended by the ATCC.

### RNA extraction and quantitative real-time RT-PCR

Total RNA was isolated from human frozen tissues using the mirVana miRNA isolation kit (Ambion, Austin, Texas, USA) according to the manufacturer's instructions. The concentration and purity of RNA were spectrophotometrically determined by measuring the optical density (A260/280>2.0, A260/230>1.8) using a NanoDrop ND-2000 spectrophotometer (Thermo Scientific Wilmington, DE, USA). cDNA was synthesized according to the TaqMan microRNA Assay protocol (Applied Biosystems) using 1 μg of total RNA as a template. qPCR analyses were conducted to quantify mRNA expression using SYBR Premix (Applied Biosystems) and GAPDH as an internal control. The sequences of the primers were as follows: FZD8 forward, 5′-GATGGGATTGCACGGTTTGG-3′, FZD8 reverse, 5′-ACCCGTATTTACGTGGGGTG-3′; GAPDHforward,5′-AATCCCATCACCATCTTCCA-3′, GAPDH reverse 5′-TGGACTCCACGACGTACTCA-3′. The TaqMan MicroRNA Assay kit (Applied Biosystems) was used to measure the levels of miR-375 after reverse transcribing with sequence-specific primers (Applied Biosystems), and U6 small nuclear RNA was used as an internal control. Real-time PCR was performed on an Applied Biosystems 7500 Sequence Detection System.

### Vector construction and cell transfection

The miR-375 expression plasmid was generated by cloning the genomic pre-miR-375 gene, flanked by a 300-nt-sequence on each side, into OriGene's pCMV6-Mir Vector to generate the plasmid pCMV-miR-375. The empty vector was used as a control. To establish a cell line that stably expressed ectopic miR-375, miR-375 expression vectors were transfected into HCT-116 cells, and the cells were selected with G418 (400μg/ml) for 3-4 weeks. Anti-miR-375, a nonspecific anti-miR-375 control, FZD8 siRNA oligonucleotides, and the control siRNA oligonucleotides were purchased from Riobio(Guangzhou, China) and transfected at a concentration of 100nmol/l using riboFECT CP Reagent (Guangzhou, China). The FZD8 expression vector was constructed by inserting the ORF sequence into the pGCL vector (GeneChem, Shanghai, China). The cells were transfected according to the manufacturer's protocol and harvested 96 hours after transfection. The reporter plasmids containing wild-type (CCTTTGATC, TOP flash) or mutant (CCTTTGGCC, FOP flash) TCF/LEF DNA binding sites were purchased from Upstate Biotechnology.

### Wound healing, cell migration and invasion assays

Cells (2×10^5^) were plated in 6-well plates and allowed to reach confluence. Streaks were created in the monolayer with a pipette tip. Migration was observed and the cells were photographed 24, 48, and 72 hours after wounding. For the migration assays, 1.2×10^5^ cells were added into the upper chamber of the insert (BD Bioscience, 8-μm pore size). For the invasion assays, 1.2×10^5^ cells were added into the upper chamber of the insert precoated with Matrigel (BD Bioscience). In both assays, the cells were plated in medium without serum, and medium containing 10% FBS was added to the lower chamber and served as chemo-attractant. After 24 hours of incubation, the cells that did not migrate or invade through the pores were carefully wiped away with cotton wool. The inserts were then stained with 10% methanol and 0.2% crystal violet, imaged, and counted under an inverted microscope (Olympus).

### Luciferase reporter assay

The pmiR-RB-REPORT Dual-luciferase miRNA Target Expression Vector (Riobio, Guangzhou, China) was used to construct the luciferase reporter vector. Double-stranded oligonucleotides containing the wild-type (wt-3′UTR) or mutant (mt-3′UTR) miR-375 binding sites in the FZD8 3′ UTR were synthesized. HCT116 CRC cells were co-transfected with the luciferase reporter vectors and the miR-375 or control vector in 24-well plates using Lipofectamine 2000 transfection reagent (Invitrogen). The indicated TOP flash or FOP flash plasmid plus 1 ng pRL-TK *Renilla* plasmid were transfected into the cells using Lipofectamine 2000 (Invitrogen). The luciferase activities were measured 48 h after transfection using a Dual-Luciferase Reporter Assay System(Promega). The *Renilla* luciferase activities were normalized to the firefly luciferase activities.

### Immunofluorescence

Cells were fixed with 4% paraformaldehyde in phosphate-buffered saline (PBS) for 15 min at room temperature. The fixed cells were permeabilized with 0.5% Triton X-100 and blocked with 5% BSA in PBS. The cells were incubated overnight with Alexa Fluor 488-conjugated monoclonal antibody against β-catenin (Cell Signaling Technology, Inc.) at 4°C. The cells were counterstained with 4′,6-diamidino-2-phenylindole(DAPI) (Calbiochem, San Diego, CA) and imaged.

### Western blot (WB) assay

Equal amounts of cell lysate were resolved by SDS-polyacrylamidegel electrophoresis(PAGE) and electrotransferred to a polyvinylidene difluoride (PVDF) membrane (Merck Millipore, Massachusetts, USA). The following primary antibodies were used: anti-FZD8 (Sigma), anti-MMP7, anti-phosphorylated β-catenin, anti-β-catenin, anti-TCF4, anti-β-actin, anti-lamin B1 (Cell Signaling Technology, Inc.). Nuclear extracts were prepared using the Nuclear Extraction Kit (Beyotime Biotechnology, Shanghai, China) according to the manufacturer's instructions.

### Immunohistochemistry

Immunohistochemistry assays for FZD8 (Aviva System Biology) were performed according to the manufacturer's instructions. The degree of immunostaining of the indicated proteins was evaluated and scored by 2 independent observers based on both the proportion of positively stained tumor cells and the staining intensities. The proportion of positively stained tumor cells was graded as follows: 0(<5%), 1(5%-25%), 2(25%-50%), 3(51%-75%) and 4(>75%). The intensity of staining was determined as follows: 0(no staining), 1(weak staining=light yellow), 2(moderate staining-yellow brown) and 3(strong staining=brown). The staining index (SI) was calculated by multiplying the staining intensity by the percentage of positive tumor cells, resulting in scores of 0, 1, 2, 3, 4, 6, 9 and 12. The cutoff values for the high and low expression of FZD8 were calculated based on a measurement of heterogeneity using the log-rank test with respect to overall survival. The optimal cutoff was identified as an SI score greater than or equal to 4, which was considered to be high expression.

### In vivo studies

We first established an orthotopic implantation murine model to examine the role of miR-375in the development and metastasis of CRC in vivo. The mice were orthotopically implanted as previously described [51]. Briefly, HCT116 cells stably expressing miR-375 or miR-NC vector (miR-negative control vector) were subcutaneously injected into 6-week-old male BALB/C nude mice. After the xenografts were established, they were excised and minced into 1-mm^3^ pieces. The pieces were then sub-serosally implanted into the cecum of other BALB/C nude mice. Twelve weeks after implantation, the animals were euthanized to collect organs. Hematoxylin and eosin(HE) staining was performed to obtain histological evidence of the tumor.

Next, we established another murine liver tumor metastasis model via spleen injection, which mimics the colonization and outgrowth phases of the tumor metastatic cascade, to further validate the role of miR-375 in CRC metastases. We injected HCT116 cells stably expressing miR-375 or miR-NC vector into the spleens of 6-week-old male BALB/C nude mice. We then used magnetic resonance imaging(MRI) to monitor tumor metastasis. After 6 weeks, mice in both groups were euthanized, and their livers were harvested. The metastases in the livers were counted, and the tumor tissues were fixed in formalin, embedded in paraffin and examined by H&E staining.

Furthermore, to test the impact of miR-375 on the survival of mice bearing tumors, we injected HCT116 cells stably expressing miR-375 or miR-NC vector into 2 groups of 6-week-old male NOD/SCID mice. The survival changes were monitored daily. After 3 months, the animals were euthanized, the peritoneal metastases were assessed, and the survival rates of the animals were calculated. All procedures were in accordance with the guidelines of the laboratory animal ethics committee of Beijing Chao-Yang Hospital, Capital Medical University.

### Statistical analysis

The data were statistically analyzed using the SPSS software package (SPSS Standard version 16.0, SPSS Inc.). The C_t_ value (C_t_) was calculated by the SDS 2.0.5 software (Applied Biosystems) using the automatic threshold setting. All real-time PCR reactions were run in triplicate, and the average threshold cycles were calculated. The average expression levels of miR-375 were normalized using U6 as a reference gene, and the 2^−Δct^ method was subsequently applied. The 2^−ΔΔct^ method was used to express the level of miR-375 in CRC tissues and matched normal mucosa samples. The significance of differences in the miR-375 levels between tumor tissues and the adjacent normal mucosa was evaluated using the two–tailed non-parametric Wilcoxon test. Differences between variables were assessed using the chi-square test or Fisher's exact test. The survival of all CRC patients was calculated with a Kaplan-Meier analysis. A log-rank test was used to compare different survival curves. A multivariate survival analysis was performed on all parameters that were found to significantly differ in a univariate analysis using the Cox regression model. The data derived from cell-line experiments are presented as the mean±SD and assessed with a two-tailed Student's t-test. *p* values<0.05 were considered to indicate significant differences.

## SUPPLEMENTARY FIGURES AND TABLES


