# Supplementary material for: MicroRNA-375 suppresses human colorectal cancer metastasis by targeting Frizzled 8
Source: Oncotarget. 2016 Jun 3;7(26):40644–56. doi: 10.18632/oncotarget.9811 (PMC5130033; doi:10.18632/oncotarget.9811)
Supplement: Supplementary file 1 [file oncotarget-07-40644-s001.pdf]

SUPPLEMENTARY FIGURES AND TABLES

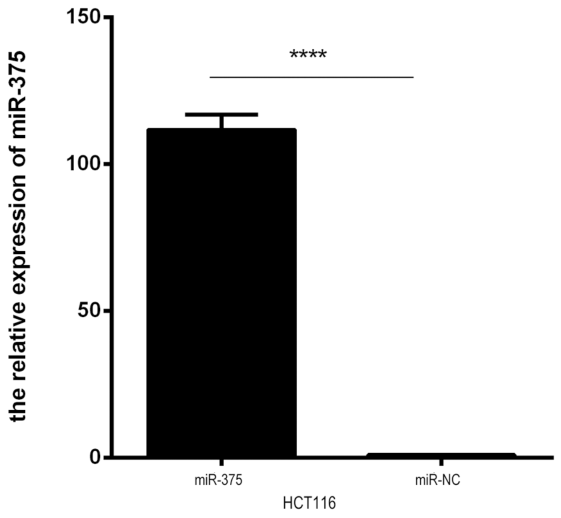

Supplementary Figure S1: The mRNA expression of miR-375 detected by quantitative reverse transcription polymerase chain reaction (qRT-PCR) in HCT116 cells stably transfected by plasmid. $p<0.0001$. The experiments were repeated at least 3 times and error bars represent mean±SD.

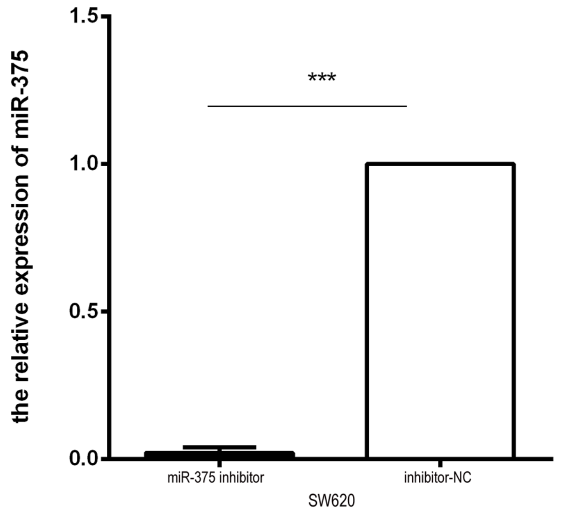

Supplementary Figure S2: The mRNA expression of miR-375 detected by qRT-PCR in SW620 cells transiently transfected with miR-375 inhibitor. $p=0.0001$. The experiments were repeated at least 3 times and error bars represent mean±SD.

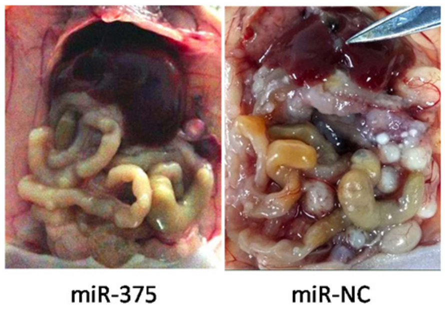

Supplementary Figure S3: The occurrence of liver metastases in BALB/C nude mice treated with HCT116 cells via spleen injection.

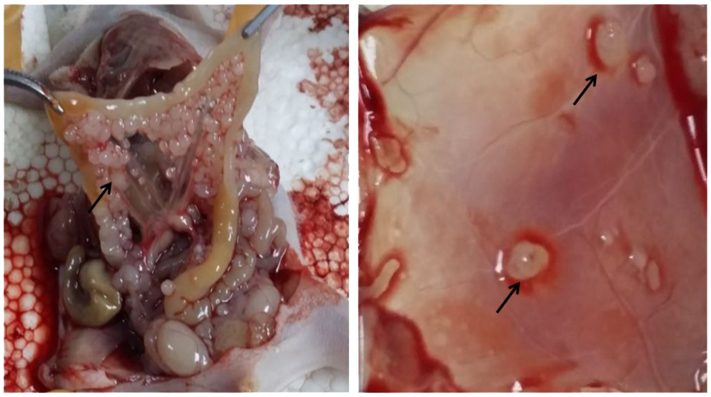

Supplementary Figure S4: The occurrence of peritoneal metastases in BALB/C nude mice treated with HCT116 cells via spleen injection for miR-NC group. No peritoneal metastases were observed for miR-375 group (Left: Peritoneal metastases×10; Right: Peritoneal metastases ×20).

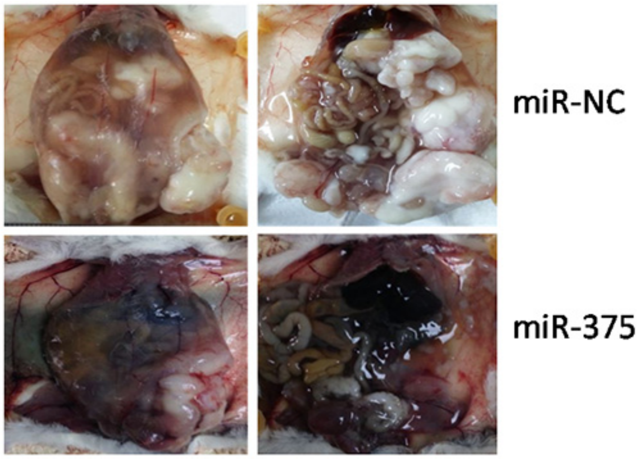

Supplementary Figure S5: The occurrence of peritoneal metastases in NOD/SCID mice treated with HCT116 cells containing miR-375 or control vector via subcutaneous injection.

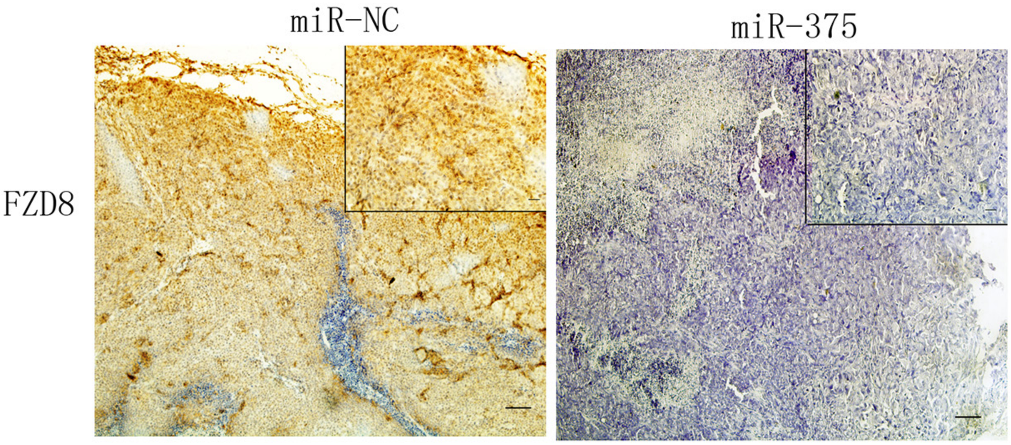

Supplementary Figure S6: IHC analysis of FZD8 protein expression in the subcutaneous xenograft of BALB/C nude mice (original magnification, ×100 and ×400).

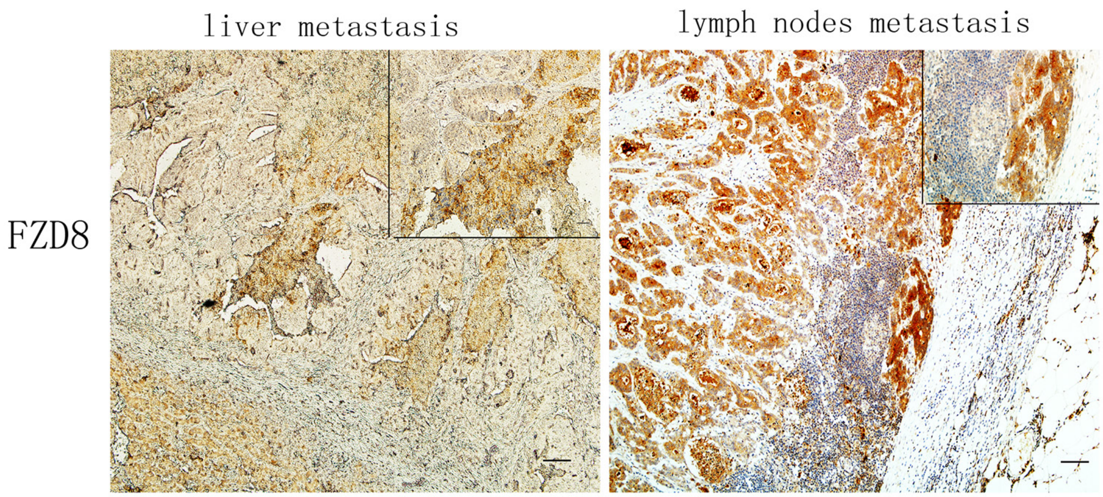

Supplementary Figure S7: IHC analysis of FZD8 protein expression in patients with liver metastasis and lymph nodes metastasis(original magnification, ×100 and ×400).

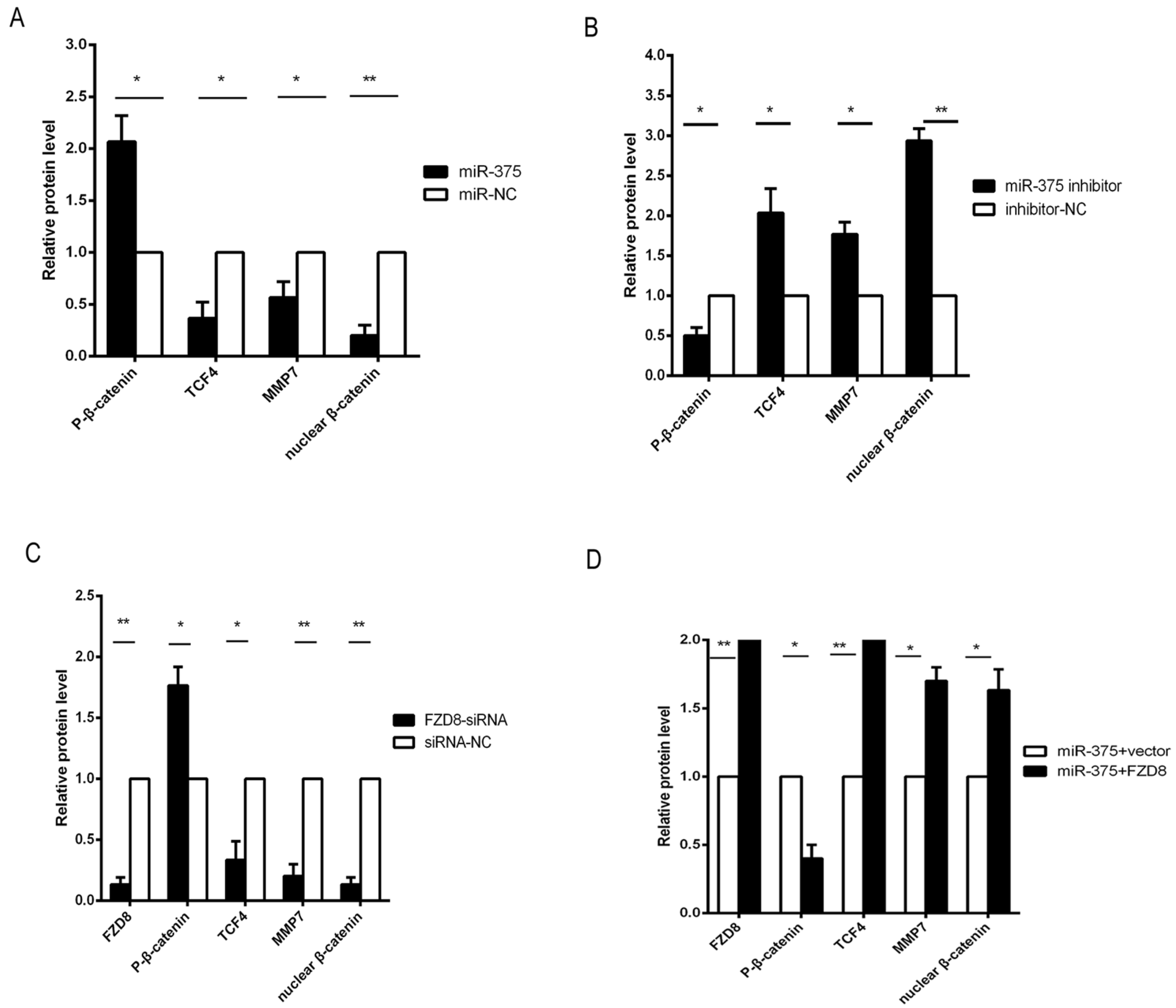

Supplementary Figure S8: WB analysis of the protein levels of FZD8, phosphorylated β-catenin protein, TCF4, MMP7 and nuclear β-catenin in indicated cells. Experiments were repeated at least 3 times.*p<0.05,**p<0.01.

Supplementary Table S1: The relationship between miR-375 expression and clinicopathological features in human colorectal cancer

Variables	Number	miR-375 expression	P value
**Age**			
≤60	36	0.14	0.35
>60	54	0.29	
**Gender**			
male	54	0.27	0.63
female	36	0.22	
**Differentiation**			
Poor+mucinous	28	0.28	0.57
Moderate+high	62	0.19	
**TNM stage**			
I+II	31	0.23	0.90
III+IV	59	0.26	
**Invasive depth**			
T1+T2	8	0.28	0.08
T3+T4	82	0.09	
**Lymph nodes**			
N0	39	0.28	0.59
N1+N2	51	0.2	
**Metastases**			
negative	76	0.44	0.55
positive	14	0.19	
**Perineuralinvasion**			
negative	59	0.26	0.83
positive	31	0.20	
**Vessel embolus**			
negative	49	0.30	0.004
positive	41	0.14	

Supplementary Table S2: The number of BALB/C nude mice developing metastases in the liver tumor metastasis model via spleen injection

	miR-375	miR-NC
liver metastases	2	4
peritoneal metastases	0	2
ascites	0	2

Supplementary Table S3: The number of NOD/SCID mice developing metastases in the subcutaneous xenograft group

	miR-375	miR-NC
liver metastases	0	1
lung metastases	0	2
peritoneal metastases	0	2

Supplementary Table S4: Comparison ofFZD8 expression in normal tissues, colorectal cancer tissues and metastases

Tissue types	FZD8 protein expression				Total number	Positive rate(%)
	-	+	++	+++		
Adjacent normal mucosa	10	39	13	1	63	22.2%$^{*\#\&}$
Colorectal cancer	4	24	30	5	63	55.6%*
Liver metastases	1	1	6	2	10	80%$^{\#}$
Lymph nodes metastases	0	3	6	1	10	70%$^{\&}$

$^{*}\chi^2$=14.72, p<0.001; $^{\#}\chi^2$=11.077, p=0.001; $^{\&}\chi^2$=7.424, p=0.006

Supplementary Table S5: The relationship between FZD8 expression and clinicopathological features in human colorectal carcinoma

Variables		FZD8expression		
**Total number**		**Low**	**High**	**P value**
		**(n=28)**	**(n=35)**	
**Age**				
≤60	20	9	11	0.952
>60	43	19	24	
**Gender**				
Male	28	16	12	0.070
Female	35	12	23	
**Differentiation**				
Poor+mucinous	9	3	6	0.717
Moderate+high	54	25	29	
**TNM stage**				
I+II	42	22	20	0.073
III+IV	21	6	15	
**Invasive depth**				
T1+T2	11	7	4	0.282
T3+T4	52	21	31	
**Lymph nodes**				
N0	43	22	21	0.116
N1+N2	20	6	14	
**Metastases**				
negative	57	27	30	0.314
positive	6	1	5	
**Perineuralinvasion**				
negative	52	26	26	0.111
positive	11	2	9	
**Vessel embolus**				
negative	55	26	29	0.422
positive	8	2	6	
